# Influence of TP53 and CDH1 genes in hepatocellular cancer spheroid formation and culture: a model system to understand cancer cell growth mechanics

**DOI:** 10.1186/s12935-016-0318-1

**Published:** 2016-06-13

**Authors:** Joseph M. Pomo, Robert M. Taylor, Rama R. Gullapalli

**Affiliations:** Department of Pathology, University of New Mexico, Room 308, MSC06-4840, Albuquerque, NM 87131 USA; Department of Chemical and Biological Engineering, University of New Mexico, Room 333A, MSC08-4640, Albuquerque, NM 87131 USA

**Keywords:** Hepatocellular carcinoma, Epithelial-mesenchymal transition, Tumor spheroids, TP53, CDH1

## Abstract

**Background:**

Spheroid based culture methods are gaining prominence to elucidate the role of the microenvironment in liver carcinogenesis. Additionally, the phenomenon of epithelial-mesenchymal transition also plays an important role in determining the metastatic potential of liver cancer. Tumor spheroids are thus important models to understand the basic biology of liver cancer.

**Methods:**

We cultured, characterized and examined the formation of compact 3-D micro-tumor spheroids in five hepatocellular carcinoma (HCC) cell lines, each with differing TP53 mutational status (wt vs mutant vs null). Spheroid viability and death was systematically measured over a course of a 10 day growth period using various assays. We also examined the TP53 and E-cadherin (CDH1) mRNA and protein expression status in each cell line of the 2-D and 3-D cell models.

**Results:**

A novel finding of our study was the identification of variable 3-D spheroid morphology in individual cell lines, ranging from large and compact, to small and unstable spheroid morphologies. The observed morphological differences between the spheroids were robust and consistent over the duration of spheroid culture growth of 10 days in a repeatable manner. Highly variable CDH1 expression was identified depending on the TP53 mutational status of the individual HCC cell line, which may explain the variable spheroid morphology. We observed consistent patterns of TP53 and CDH1 expression in both 2-D and 3-D culture models.

**Conclusions:**

In conclusion, we show that 3-D spheroids are a useful model to determine the morphological growth characteristics of cell lines which are not immediately apparent in routine 2-D culture methods. 3-D culture methods may provide a better alternative to study the process of epithelial-mesenchymal transition (EMT) which is important in the process of liver cancer metastasis.

## Background

The incidence of hepatocellular carcinoma (HCC) has been steadily increasing in the past decade. HCC is currently one of the fastest growing cancers in the United States. The outcomes in cases of HCC are dismal, with a 5 year survival of less than 8 % for stage III and above [1, 2]. Globally, HCC is the fifth most common cancer and the third most deadly [2]. The majority of cases of HCC globally are due to Hepatitis viral infections, namely, Hepatitis B and C [2]. In the United States, the major cause of HCC is due to alcoholism, however, obesity is also a fast emerging risk factor in the United States [3, 4]. The tumor microenvironment plays an important role in modulating HCC tumor biology, as well the efficacy of chemotherapeutic treatments.

Traditional research techniques of cancer biology utilize two-dimensional, monolayer cell cultures grown in flasks. However, the role of the spatial cell signaling cues of the tumor microenvironment in the formation and metastasis of tumors, including HCC, is being increasingly appreciated [5, 6]. Previous studies have noted differences in the gene expression patterns, morphological features, cell growth kinetics and metabolic rates of 3-D tumor spheroids compared to 2-D monolayer cultures [5]. 3-D tumor spheroids allow a controlled, stratified representation of the tumor microenvironment to mathematically model the influence of external perturbants such as chemotherapeutic drugs which are affected greatly by the tumor microenvironment [7]. It has been previously noted that 3-D representations of tumor spheroids are more resistant to radiotherapy and chemotherapy than 2-D cell cultures [7, 8]. This is similar to the patterns of radio and chemo-resistance noted in real clinical patient tumors. Thus, tumor spheroids represent a valuable model to examine issues related to radio and chemo resistance in patients. Additionally, it is possible to create complex tumor spheroid models by incorporating other cellular components such as fibroblasts or macrophages to create realistic models of the in vivo tumor microenvironment [9].

The role of adhesion molecules such as integrins and E-cadherin (CDH1), in intra-cellular cancer cell signal transduction have been elucidated previously, including HCC [10–13]. The presence of cell–cell contacts as well as cell–matrix contacts play a crucial role in modulating the morphological and gene expression patterns of the cancer cells [10, 12]. The extra-cellular matrix (ECM) of a tumor microenvironment is composed of various glycoproteins and fibrous proteins which allow cancer cells to interact with each other, as well as normal cells [12]. The precise mechanism of the nature of interactions remains unclear.

In the current study we describe the growth and characterization of tumor spheroids from HCC cell lines. We have optimized the growth conditions of five HCC cell line spheroids (C3A, HepG2, Hep3B, SNU-387 and SNU-475) that are used in our lab. The initial choice of cell lines was driven by the TP53 mutational status of in each cell line. TP53 gene somatic mutation was observed in HCC in approximately ~21 % of clinical cases in one study [14]. In general, TP53 somatic mutations tend to portend worse clinical outcomes for cancer patients due to its’ central role as a tumor suppressor gene. There is a crucial need to understand the precise role played by TP53 in the pathogenesis of HCC and other forms of cancer. In the current paper, we have systematically explored the optimal conditions required for the growth of these HCC tumor spheroids in a reproducible manner. We then proceeded to examine the growth morphology of each cell line. We also evaluated gene expression patterns in 2-D and 3-D with a focus on TP53 and CDH1 in the chosen cell lines. CDH1 is localized to the surface of growing cancer cells and plays a major role in the formation of cell–cell contacts and is responsible for the maintenance of the cell polarity [10]. In addition, CDH1 plays an important role in the phenomenon of EMT [10, 15]. We plan to utilize the 3-D tumor spheroid model to elucidate the detailed molecular mechanisms of the phenomenon of EMT in liver cancer in the future.

## Results and discussion

### Establishing and characterizing the optimal growth conditions of the tumor spheroids

**Fig. 1:**
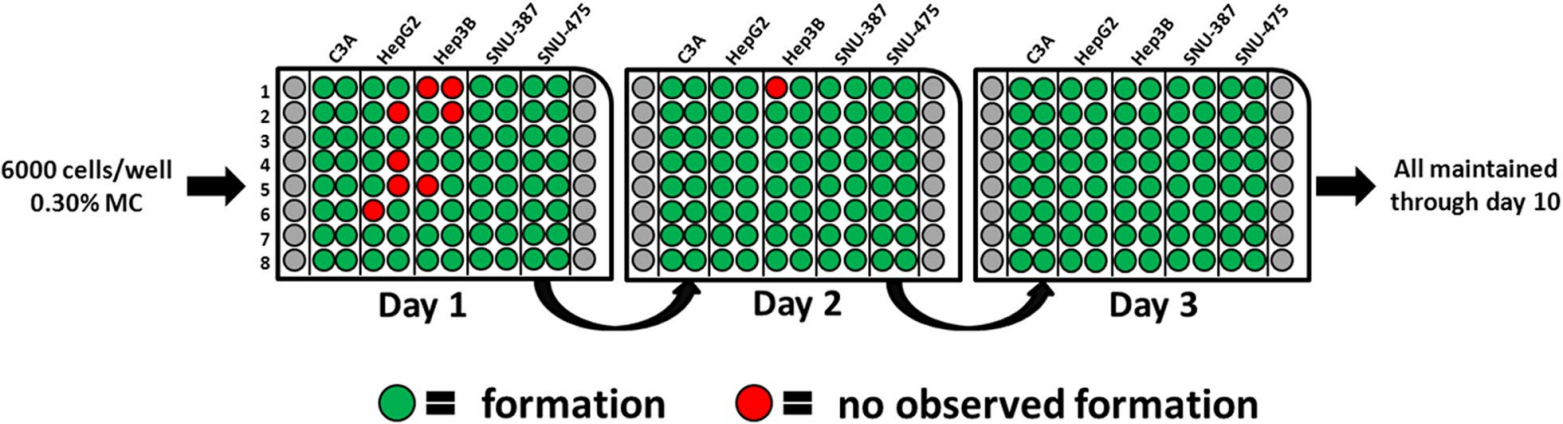
Spheroid growth methods: spheroid growth was recorded at 24 h intervals. Formation was successful in all five cell lines with at least 7 of 8 wells developing structures within 24 h of incubation. Media changes were performed on every other day according to droplet volume

Hanging drop plates (HDPs) suspend tumor cells in a droplet of media which encourages cell–cell interactions in three dimensions resulting in the formation of a spheroid [7]. We elected to use HDPs for tumor spheroid formation due to the ease of cell seeding, spheroid imaging and capture, as well as compatibility with fluorescent plate readers and high-throughput, automated, liquid handling equipment for future studies. Numerous preliminary trials were conducted to determine the optimal conditions for spheroid growth of the HCC cell lines. An important factor in the successful growth of spheroids as hanging drops is the composition of the drop solution itself and its ability to form a support matrix to facilitate cell–cell interactions. For each cell line, we used the native recommended media supplemented with 10 % FBS (EMEM & RPMI) according to ATCC recommendations. For the support matrix, we investigated the addition of 0.30 % Cultrex^**®**^ Basement Membrane Extract or Methylcellulose (MC) at 0.30 % in the initial trials. The protocol for preparation of MC stock was adapted and modified from [5]. MC and BME supplementation were subsequently tested at 0.15, 0.30 and 0.50 % by droplet volume at varying seeding cell densities of 3000, 6000 and 20,000 cells/well (data not shown). It is important to note that we achieved spheroid formation at all the cell seeding densities and MC concentrations, indicating the robustness of the HDP method. Our goal, however, was to create a consistent method to form individual, compact tumors. BME supplementation was a slight disadvantage in this regard because it created dispersed ‘compartments’ within the media drop which housed small cell aggregates, instead of one compact sphere. In our final analysis we determined that 6000 cells/well with a supplementary concentration of 0.30 % MC, was ideal for consistent spheroid formation for the HepG2, C3A and Hep3B cell lines (P53-wt and P53-Null). In the case of the TP53-mutant phenotype cell lines (SNU-387 and SNU-475), we observed inconsistent spheroid morphology patterns in our initial experiments. We suspected a molecular level phenomenon in these TP53 mutant cell lines as the root cause of this issue (described below). Increasing the viscosity of the medium (1 % MC instead of 0.3 % MC) ensured optimal, spherical growth morphology of the SNU-387 and SNU-475 cell spheroids. We hypothesized that the environmental vibrations were the cause of the poor spheroid morphology in these cell lines. By increasing the viscosity of the surrounding medium, the effect of these vibrations on tumor spheroid formation can be minimized in these cell lines. Tumor spheroid counts were conducted over duration of 10 days incubation (Fig. 1), and it was shown that all the methods produced long-lasting spheroid structures. MC was ultimately chosen for culturing in this study based on the ease of use, availability in large quantities and low costs—compared to commercially produced BME supplementation matrix. MC itself does not act as an extra-cellular matrix, but instead creates a polysaccharide based viscous environment which facilitates cell–cell interactions via cell junction formation.

### The tumor spheroids show decreasing viability with increasing length of spheroid culture

We sought to establish the spheroid viability of the HCC cell lines throughout the 10 day culture period. We used three, commercially available assays to establish the viability of the tumor cells in these spheroids: (a) alamarBlue^®^ cell proliferation assay (b) CellTiter-Glo^®^ luminescent assay and (c) LDH cytotoxicity assay.

**Fig. 2:**
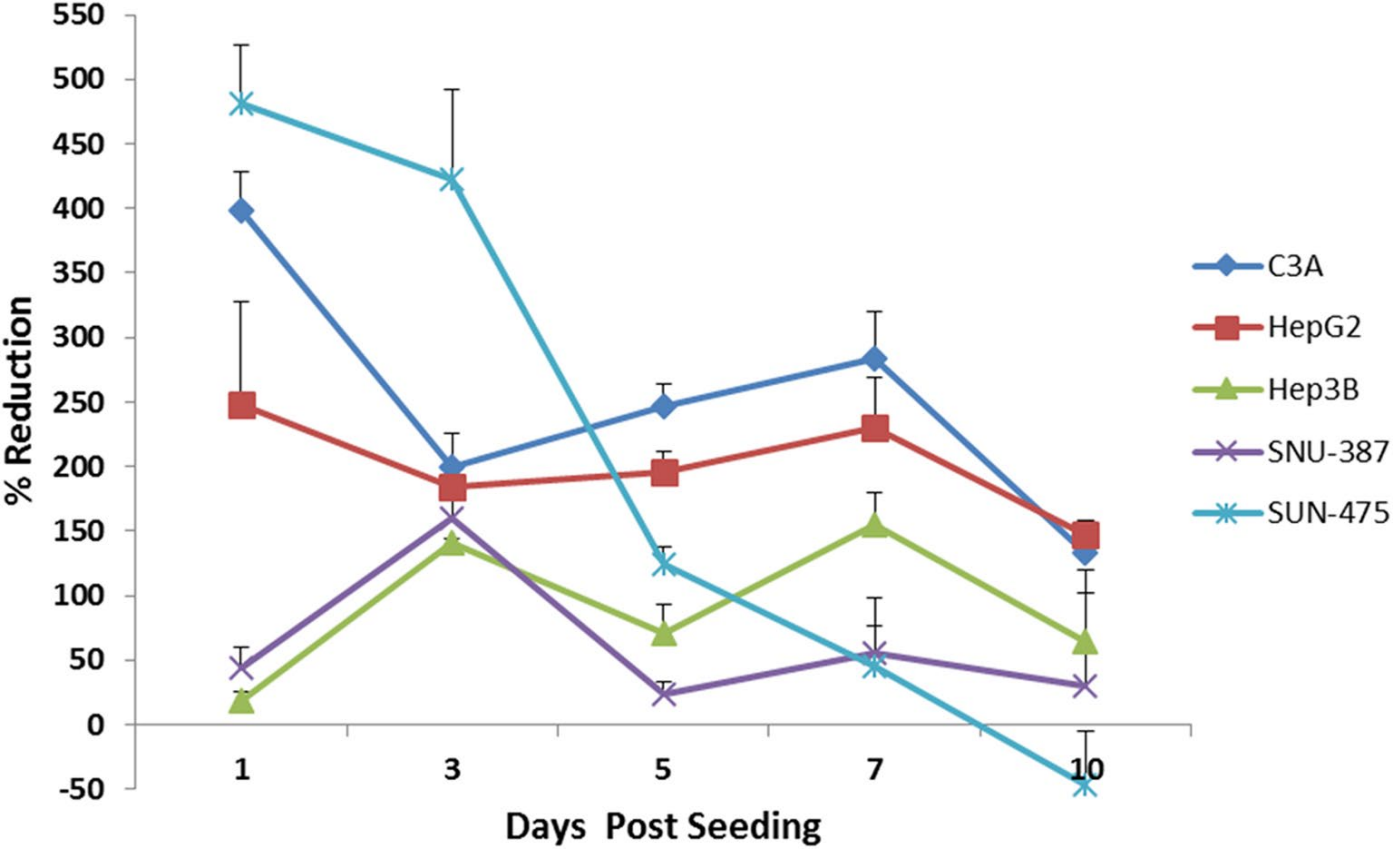
Percent reduction of alamarBlue^®^ reagent: absorbance and emission (535/595 nm) of cell culture media was measured on days 1,3,5,7 and 10 after seeding. Higher percentage reduction indicates more metabolically active cells. Most cell lines show an overall decreasing trend in % reduction indicating the presence of fewer live cells within cultures over time. The slight increase in % reduction found among the wt (day 3–7), null (day 5–7) and SNU-387 (day 5–7) cell lines may be attributed to active remodeling in which necrotic cells are shed from the tumor surface by live cells

AlamarBlue^**®**^ is a widely-used cell proliferation indicator which works by estimating the reductive and oxidative components of the media in which the cells are being cultured. As the tumor spheroid cells grow, innate metabolic activity results in a chemical reduction of alamarBlue^®^. Continued growth maintains a reduced environment while inhibition of growth leads to an oxidized environment. HCC lines produced absorbance values which were between 95 % and just over 200 % greater compared to control wells containing media only (Fig. 2). In Fig. 2, we note that all the five cell lines maintained a reductive capability at the end of the 10 day growth period indicating spheroid growth viability at the end of the chosen testing period. This is not surprising as it has been observed in other studies that the spheroids continue to grow for as long as 20-25 days before cell necrosis sets in a centrifugal fashion (inside out), reducing the overall viability of the spheroids [16]. On day 5 of growth, we observed the largest alamarBlue^®^ reductions in the TP53 wild type cell lines (HepG2–195 % and C3A–247 %) and the TP53-mutant cell line SNU-475 (124 %) and the lowest reductions in the TP53-null cell line Hep3B and the remaining TP53-mutant, SNU-387 (71 and 23 %, respectively). It is important to note that the alamarBlue^®^ assay measures the redox nature of the “cell media” of the spheroids and not the viability of the cells themselves. Our current results indicate the presence of a “growth state” in all the cell lines at the end of 10 days of spheroid culture used in this study.

**Fig. 3:**
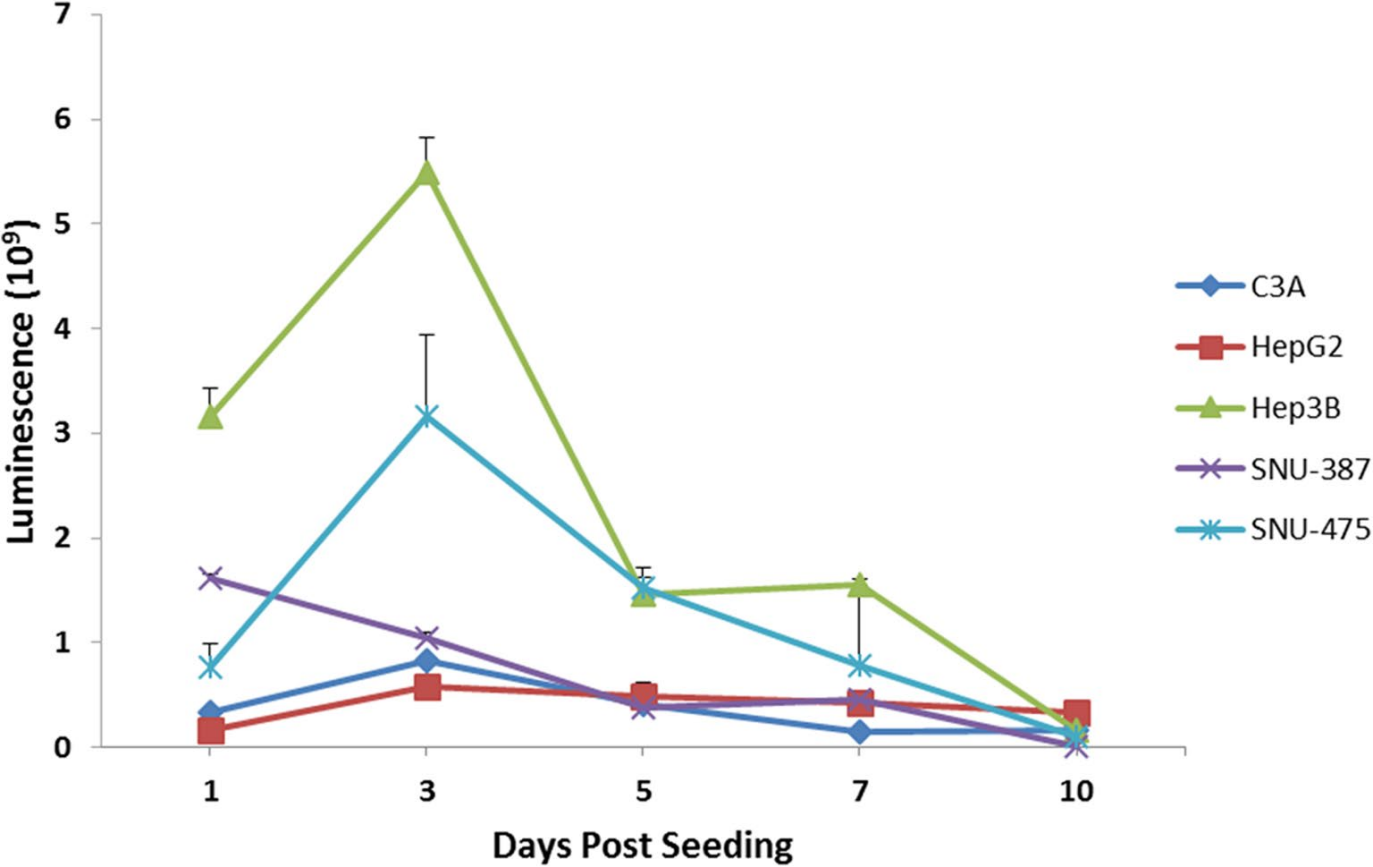
CellTiter-Glo^®^ viability: measures ATP Luminescence indicative of active metabolism within the cells. There is an increase from days 0–3 during the active remodeling phase followed by a reduction from days 3–5 during the compaction phase. Subsequently, from days 5–10, there is a progressive decrease indicating reducing viability in all cell lines. Luminescence values are normalized to the total spheroid volume

To measure the viability of the spheroid cells themselves in a direct manner, we performed the CellTiter-Glo^®^ assay as a complement to alamarBlue^**®**^. The CellTiter-Glo^®^ assay is a highly sensitive assay which determines the number of viable cells based on a quantitation of the ATP present in the cells using a luciferase based reaction. The amount of ATP present in the cells indicates their metabolic activity. The HCC cell lines in the current study generate different sized spheroids (see ‘‘Results’’ section). To account for the variability of the number of cells in each spheroid cell line, we normalized the CellTiter-Glo^®^ assay values to the average volume of the spheroid (Fig. 3). In all of the cell lines except SNU-387, we observe an initial increase the amount of ATP within the first 72 h after seeding the HDP. Subsequently, on days 3–5, we notice a steep drop in the amount of ATP generated in each cell line. Over the next 5 days until day 10 of culture, we notice a progressive decrease in the amount of ATP present in all the cell lines. At the end of day 10, we notice the lowest amount of ATP in all the cell lines indicating a progressive loss of viability of the cells in the spheroid over the duration of the growth of 10 days. Interestingly, we notice the maximum increase and decrease of ATP values in the TP53-null cell line (Hep3B) indicating a high degree of metabolic activity within this cell line.

**Fig. 4:**
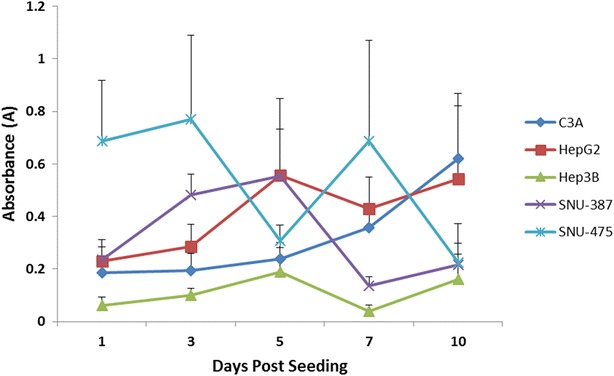
LDH absorbance: higher LDH absorbance values indicate a larger proportion of damaged cells in the sample. Similar to the CellTiter-Glo^®^ assay, there is an increase in values from days 1–3 during the active remodeling phase, which continues and stabilizes during the compaction phase on days 3–5. Subsequently, there is an increasing trend of values from days 5–10 unlike the CellTiter-Glo^®^ assay (Fig. 3)

To assess the amount of cytotoxicity and cell death in the spheroids models, we used a lactate dehydrogenase (LDH) enzyme assay. Cells undergoing active remodeling or cell death release lactate-dehydrogenase enzyme into the culture media which can then be measured using a plate reader set-up. The amount of LDH measured is indicative of the extent of cell death in the measured sample. The LDH assay (cell death measurement) is complementary to the CellTiter-Glo^®^ and alamarBlue^®^ assays (live cell measurement). Similar to the CellTiter-Glo^®^ assay, we normalized the LDH values by obtaining measurements from a single average volume (~15 uL) of the media in each of the HCC cell lines. We observe a progressive increase in LDH values in the initial 3–5 days in all the cell lines (Fig. 4) indicative of an active metabolic and remodeling process. From days 5–7, we notice a slight decrease in the LDH absorbance of most lines, with the exception of C3A and SNU-475. Finally from days 7–10, all of the cell lines (except SNU-475) show a steady increase in the LDH absorbance indicating a progressive loss in spheroid viability during this period. The LDH results observed are complementary to the results seen from the CellTiter-Glo^®^ assay which shows a progressive decline in luminescence on days 5–10 (Fig. 3), whereas the LDH assay shows an increase in the absorbance values during the same time period. The measured LDH values of the TP53-mutant SNU-475 cell line are highly variable which we think may be due to the highly aggressive and metabolically active nature of this line secondary to the double TP53 mutation present in this cell line [17].

Overall, these results appear to indicate 3 distinct phases of tumor spheroid growth formation. In the first 72 h after seeding, there is extensive growth and remodeling resulting in the increase of the CellTiter-Glo^®^ and LDH values simultaneously (phase 1). Days 3–5 constitute phase 2, which is characterized by consolidation of the spheroid growth, while days 5–10 (phase 3) are indicated by progressive decline in tumor spheroid viability in all of our cancer cell lines. Day 5 post seeding appears to be the most stable with regards to spheroid metabolic activity and the stability of the spheroid size. Based on the results of these assays, we conclude that the days 4–5 post-seeding is the appropriate window period to conduct functional assays of the tumor spheroids in all the 5 HCC cell lines.

### Tumor spheroids show variable morphologies and sizes in three dimensions depending on the TP53 mutational status

One of our initial observations during the testing phase of the spheroid growth protocol was the distinct differences of the spheroid morphology we noted between the five HCC cell lines included in this study. These 3-D morphological structure differences are not immediately apparent in regular 2-D flask cell culture. Our initial observations regarding cell line specific spheroid morphology appeared to be consistent and repeatable. In order to assess these differences more rigorously, we measured multiple replicates eight of the individual spheroids for the duration of growth (10 days) and have reported the mean diameters (µm).

**Fig. 5:**
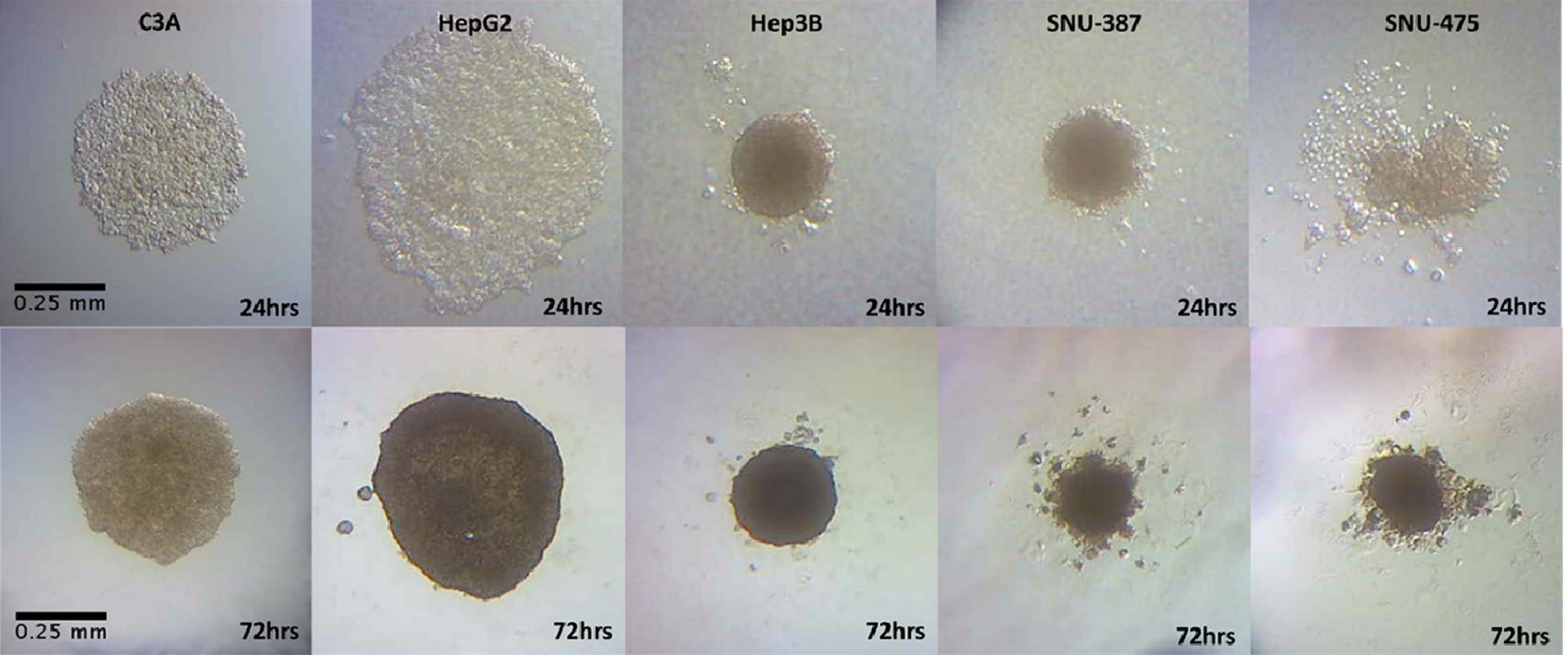
Live growth capture: *spheroids* in all 5 lines imaged at 1 and 3 days post seeding. The spheroid morphologies are distinct depending on the TP53 status. The TP53-wt cell lines, C3A and HepG2 form large *spheroids* with a smooth boundary edge. TP53-null cell line of Hep3B forms compact, intermediate-sized *spheroids*. The TP53-mutant cell lines, SNU-387 and SNU-475, form small sized *spheroids* with a rougher boundary edge. The size and shape patterns are consistent over the 10 day incubation period in multiple replicates

Qualitatively, we observe distinct differences in the shape and appearance of the individual spheroids depending on the TP53 status (see Fig. 5). The wild type TP53 cell lines of HepG2 and its derivative cell line, C3A, show the growth of large-sized spheroids with a smooth and flattened edge boundary. The TP53-null cell line Hep3B showed small and compact spheroid morphology with minimal roughness of the edge. The TP53-mutant cell lines of SNU-387 and SNU-475 showed an intermediate size characteristic with an irregular edge formation. The roughness characteristic of the spheroid edge of SNU-387 was much more predominant compared to the SNU-475 cell line. However, the approximate sizes of the TP53-mutant cell lines were comparable to each other (see Fig. 5).

**Fig. 6:**
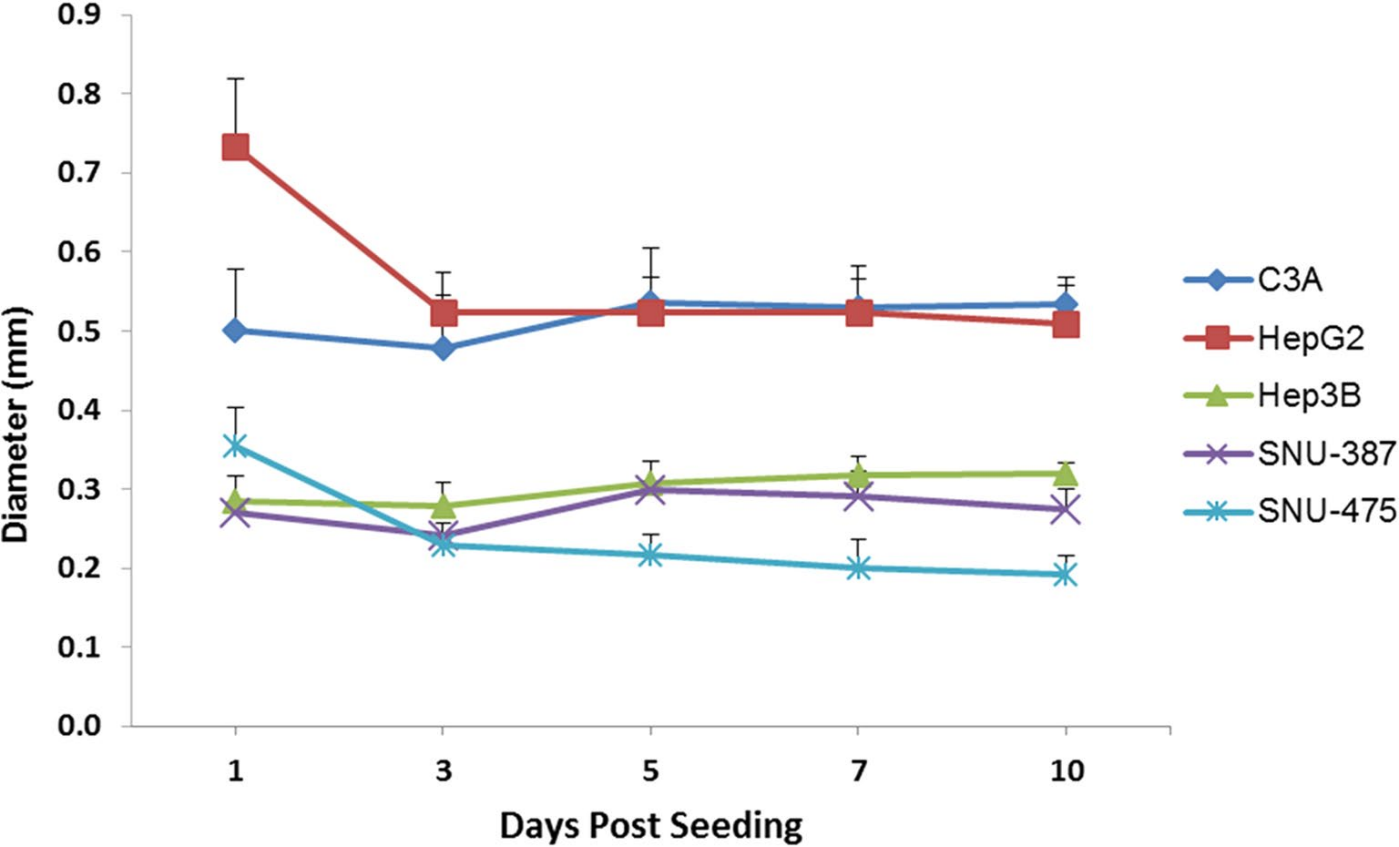
Spheroid size determination: average diameters for *spheroids* over the 10 day incubation period. Values are obtained over an average of 8 different spheroid measurements on each day. All *spheroids* show a reduction in size from day 0–3. Beginning on day 5, the size stabilizes in each cell line with minimal change by day 10

Quantitatively, we measured the diameters of multiple spheroids on days 1,3,5,7 and 10 of the spheroid growth (see Fig. 6). Within the first 72 h after seeding in the HDP, the cells aggregate to form the spheroid morphology in all the five cell lines tested. The process of compaction is finished in the first 72 h after seeding resulting in a spherical morphological appearance. Subsequently, from days 3 to 5, there appears to be a slight increase in the diameter of the spheroids in all of the cell lines during the consolidation phase (except SNU-475). From days 5–10 of spheroid growth, all the cell lines appear to maintain the size and morphology at a steady rate. The TP53-mutant cell line of SNU-475 appears to lose radius beyond day 3, though the size reduction is very minimal and within the limit of error (see Fig. 6). The average diameters of the TP53-wt cell lines on day 5 were 535.7 ± 69.49 and 522.9 ± 44.12 µm for the C3A and HepG2 cell lines respectively. The TP53-null cell line, Hep3B, had a diameter of 307.1 ± 28.81 µm. The TP53-mutant cell lines had diameters of 298.6 ± 10.95 µm (SNU-387) and 217.1 ± 25.03 µm (SNU-475). Of note, the diameter measurement of SNU-387 was difficult due to the rough boundary edge. The spheroid volumes were extrapolated from the diameter measurements and are consistent with the diameter measurements (data not shown).

### The spheroid morphologies may be explained by variable levels of CDH1 expression in individual cell lines

In order to understand the molecular level changes which may explain the varying spheroid morphologies, we examined candidate molecules in all the cell lines of this study. Specifically, we examined the TP53 gene and the E-cadherin (CDH1) gene expression status in these HCC cell lines. Cell–cell junctions play a very important role in the modulation of the tumor microenvironment [13]. There are three major types of cell junctions (a) adherens junctions/desmosomes/hemi-desmosomes (b) gap junctions and (c) tight junctions. Of these three, the adherens junctions are most well characterized in epithelial cells [13]. CDH1 is a major transmembrane protein belonging to the cadherin family involved in the formation of the adherens cell–cell junctions [13]. CDH1 gene expression is present in normal as well as tumoral liver cells at widely differing levels and is thus, a natural candidate for further study to explain the varying spheroidal morphological differences described in the previous section. We examined the TP53 and CDH1 expression differences in all five cell lines using immunofluorescence, real time-PCR and Western blotting methods.

#### Immunofluorescence staining

**Fig. 7:**
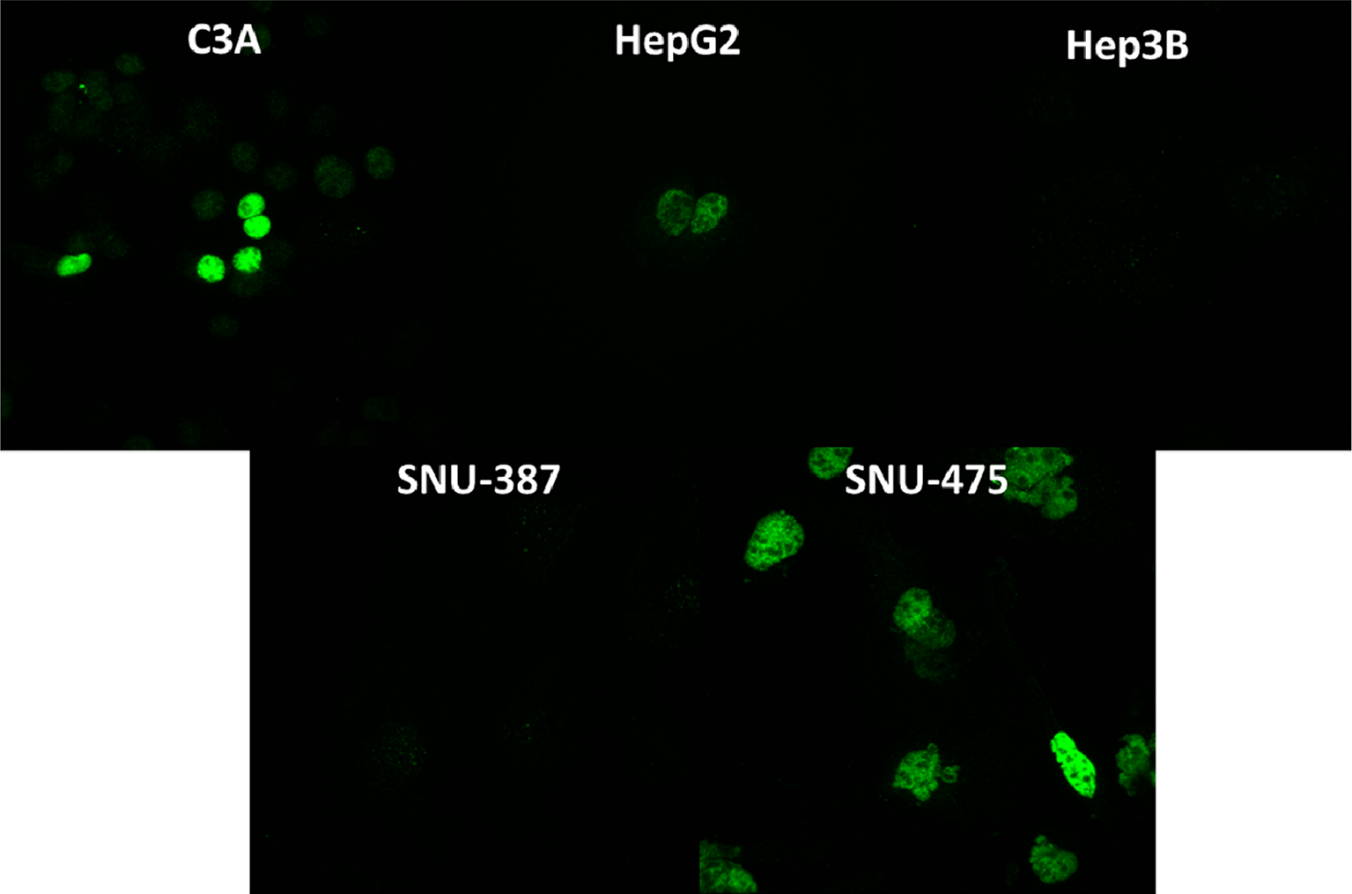
TP53 immunofluorescence: staining performed with D0-1 TP53 primary antibody and Alexa Fluor^®^ 488 secondary antibody. The TP53-wt lines, C3A and HepG2 show intermediate levels of staining. Minimal to no fluorescence was observed in TP53-null Hep3B and TP53-mutant SNU-387. The TP53-double mutant, SNU-475 showed high nuclear staining. Axioskop imaging was set at an exposure of 500 ms using a ×63 oil objective for all images

**Fig. 8:**
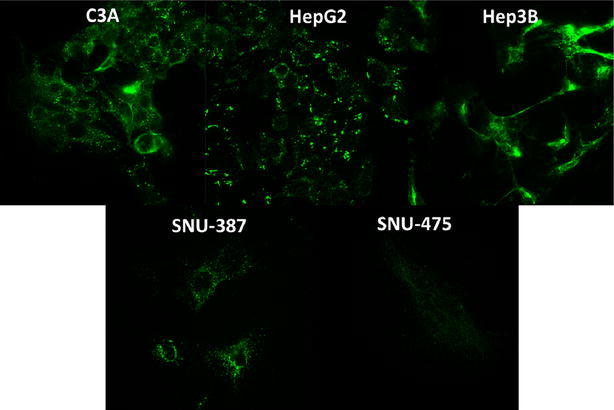
CDH1 immunofluorescence: staining performed with anti-E-cadherin primary antibody and Alexa Fluor^®^ 488 secondary antibody. E-cadherin staining was clearly more pronounced in C3A, HepG2 and Hep3B lines. The Hep3B staining appeared to be intense and localized to the surface of the cells. Minimal, dispersed staining was observed in TP53-mutant cell lines, SNU-387 and SNU-475

We performed immunofluorescence (IF) staining based on the protocol described in the methods for TP53 and CDH1 proteins. All the cell lines showed TP53 and CDH1 staining, with variable intensities (see Figs. 7, 8). For the wild-type TP53 cell lines, HepG2 and C3A showed intermediate levels of staining intensity within the nucleus of each cell. Hep3B, did not show any staining as expected due to its TP53-null status. For the TP53-mutant cell lines, the SNU-475 cell line showed an excess of staining in the nucleus while the SNU-387 cell line showed a minimal intensity of staining. Mutant proteins of TP53 are well known to accumulate within the nucleus depending on the specific nature of the mutation within the TP53 protein. In particular, mutant TP53 proteins are known to exhibit a dominant negative effect which may lead to abnormal accumulation with the nucleus. However, the dominant negative effect is usually a function of the specific position of the mutation within the protein. In contrast to SNU-475, the second TP53 mutant cell line of SNU-387 showed minimal IF staining. It is important to note that these two cell lines originated from two different individuals with mutations located in different locations of the TP53 protein [17]. An absent dominant negative effect in the mutant TP53 protein may explain the lack of IF staining in the SNU-387 cell line due to lack of nuclear accumulation.

#### Real-Time PCR data

**Fig. 9:**
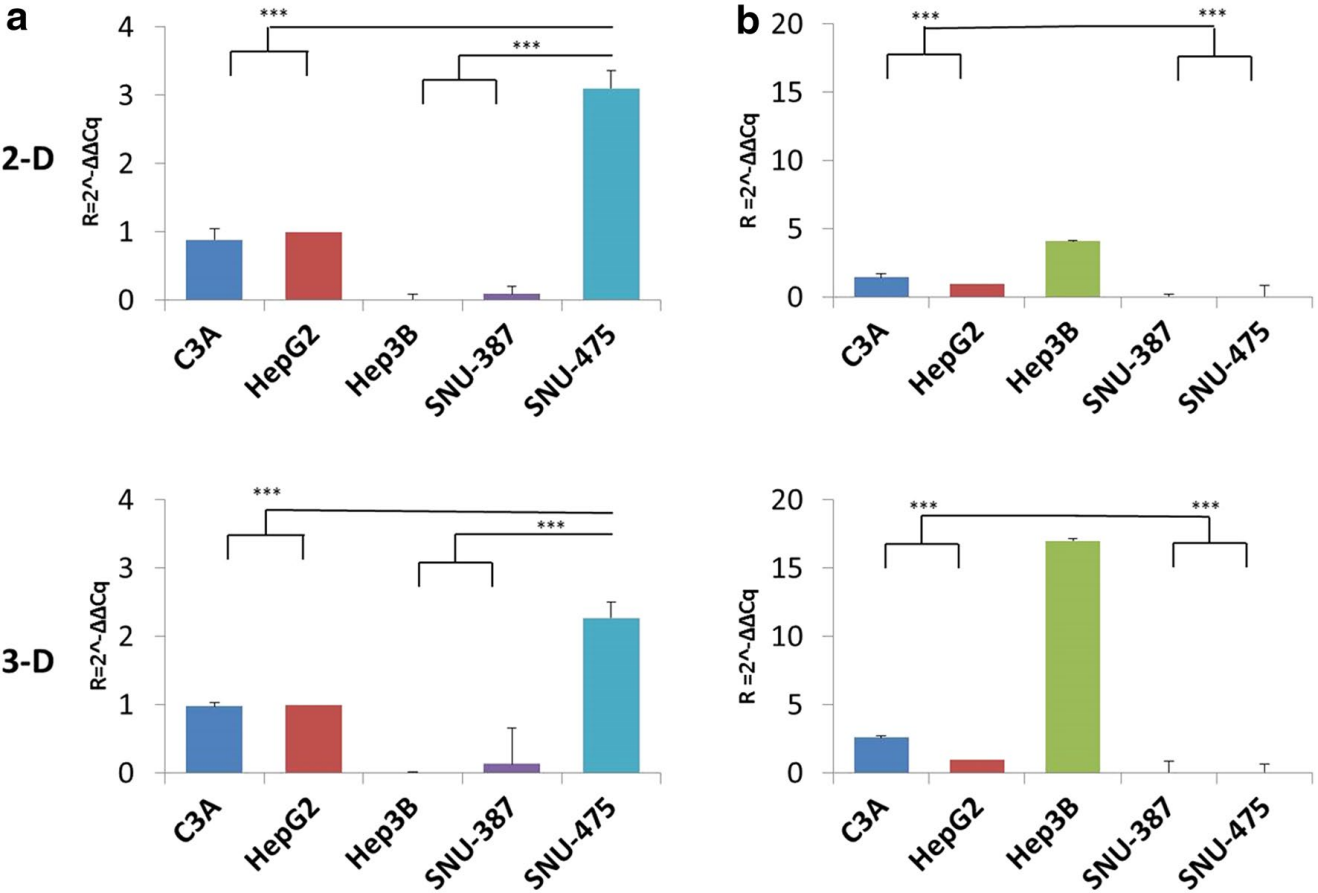
Quantitative real-time PCR: mRNA gene expression of TP53 and CDH1 genes in all five cell lines. Relative expression normalized to the value of the HepG2 (wt) cell line. **a** TP53 gene expression: The TP53-wt cell lines, C3A and HepG2 show intermediate levels of expression. There is overexpression of TP53 in the SNU-475 mutant cell line (see text). Minimal to no expression is observed in SNU-387 and TP53-null Hep3B cell lines. Similar patterns of expression is observed in both 2-D and 3-D cell cultures. **b** CDH1 gene expression: The TP53-null cell line, Hep3B shows maximal expression of CDH1 mRNA in 2-D and 3-D cultures. The TP53 wild-type cell lines show intermediate expression, while the TP53-mutants show minimal to absent expression of CDH1. ***indicates a significant difference between the compared cell lines at a 99 % level statistical significance using a paired *t* test (P-value ≤ 0.01)

We used real time-PCR analysis to corroborate the IF data described above with the appropriate controls and replicates (see methods). We conducted RT-PCR assays on cells grown in 2-D (cell culture flasks) as well as 3-D (tumor spheroids from HDP). We observe a similar pattern of RT-PCR results for the TP53 gene expression as observed for the IF staining. SNU-475 shows the highest amount of TP53 relative expression which is statistically significant compared to the wild-type cell lines of HepG2 and C3A (see Fig. 9). As expected, the Hep3B cell line does not show any TP53 gene expression due to the null status of the gene, while the SNU-387 shows a minimal level of gene expression. Interestingly, we do not observe a significantly different pattern of TP53 gene expression when comparing the 2-D cells to the 3-D tumor spheroid cells data.

For the CDH1 gene, we observe a similar pattern of gene expression as the IF staining. The TP53-null cell line of Hep3B shows the maximum expression quantitatively, while the TP53-wt cell lines (HepG2 and C3A) show an intermediate expression and the TP53-mutant cell lines (SNU-387 and SNU-475) show the least amount of expression in the cells. Interestingly, we observe a higher level of relative expression in the TP53-null cell line, Hep3B, in the 3-D tumor spheroids compared to the 2-D cell culture. This was confirmed by repeating the experiment multiple times. This overexpression of CDH1 may explain the relatively smaller size of Hep3B spheroids as well as the high degree of compactness (see Fig. 5). Previous data indicates there is a genomic level defect in the Hep3B cell line which leads to a deletion beyond exon 7 of the TP53 gene in this cell line [18]. The precise nature of the regulatory mechanism of the TP53-wt, null and mutant phenotype involved in the CDH1 gene expression is the focus of future studies within the lab.

#### Western blotting data

**Fig. 10:**
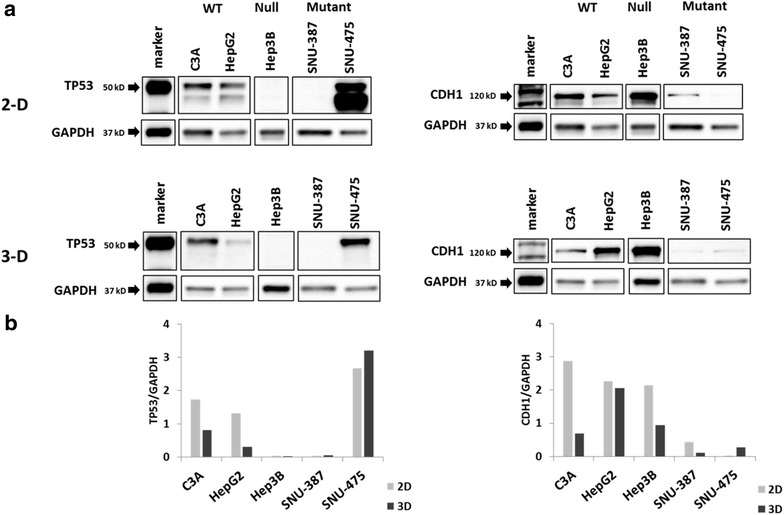
Western blotting in 2-D and 3-D cell cultures: **a** maximal TP53 protein expression is noted in TP53 mutant SNU-475 while maximal CDH1 expression is observed in the TP53-null cell line, Hep3B (see text for explanation). The patterns are consistent in 2-D and 3-D cultures. An additional band is observed in SNU-475 in 2-D which probably represents the β and γ isoforms of TP53 corresponding to 48 and 47 kDa sizes respectively. GAPDH was used a loading control. **b** Band intensities for TP53 (*left*) and CDH1 (*right*) normalized to corresponding GAPDH control. 2D and 3D ratios show some slight variation due to the inherent variability associated with quantification of western blot intensities

We examined the protein level expression patterns in the five cell lines of 2-D and 3-D culture to verify the existence of a protein-level phenomenon. The Western blot data is in concordance with the RT-PCR results and we observe the same pattern of expression at the protein level for the TP53-wt, null and mutant cell lines (see Fig. 10). An interesting observation is the presence of an additional lower-weight band in the TP53-mutant (SNU-475) when targeted with the TP53 specific antibody, DO-1 (see Fig. 10). The antibody DO-1 targets the 22–26 amino acids of the *N*-terminus of the TP53 protein. TP53 protein is well known to contain nine different isoforms which appear to be expressed at varying levels in different cancer cell lines [19]. In this instance, we believe the lower-weight band represents the β and γ isoforms of the TP53 protein which are likely overexpressed in the TP53 mutant SNU-475 cell line due to the dominant negative effects of TP53 protein. The β and γ isoforms have a molecular weight of 48 and 47 kilo Daltons (kDa) respectively, compared to the α form (~53 kDa) which would account for the location of the smaller band. No similar bands are seen in all of the other cell lines, including the other TP53 mutant cell line, SNU-387. These isoforms also probably account for the increased immunofluorescence in the SNU-475 cell line (see Fig. 7). Interestingly, this strong, isoform band observed in 2-D culture is absent from our 3-D spheroids, though the true cause is unclear. The CDH1 gene follows a similar expression pattern as the real-time PCR data in the 2-D and 3-D cell models. The overexpression of CDH1 protein in the TP53-null cell line observed at an mRNA level and discussed in the previous section is confirmed at the protein level as well. Variable level of CDH1 expression was noted in the different 3-D tumor spheroids compared to the 2-D cell culture flasks, which was also noted on RT-PCR. However, the general trends of TP53 and CDH1 protein expression are similar in the 2-D and 3-D growth methods in the study.

## Conclusions

Novel techniques for culturing cells in 3-D are of increasing importance as we identify the limitations of traditional 2-D culture in enabling our understanding of the role of the tumor microenvironment in cancer biology. The phenomenon of epithelial-mesenchymal transition (EMT) has been shown to play an important role in the pathogenesis of liver cancer by influencing the local growth and formation of distant metastasis within these tumors [20]. The phenomenon of EMT is a complex one, characterized by a multitude of changes in the tumor at a molecular level [15]. These include changes in expression patterns of nuclear transcription factors, intermediate signaling molecules and peripheral effector molecules such as CDH1 [15]. A precise understanding of the molecular interplay is crucial in order to predict the outcomes in a tumor as well as identifying potential targets for personalized therapies.

In our study we have demonstrated a feasible and consistent system for culturing hepatocellular carcinoma cell spheroids in five different cell lines (HepG2, C3A, Hep3B, SNU-475 and SNU-387) using the hanging drop method. We have characterized in detail, the necessary growth conditions for these cell lines as well as the growth viability over the duration of the growth (10 days). An extended growth period beyond 10 days is indeed feasible and may be of interest to understand issues related to hypoxia and growth necrosis in tumor spheroids. Through the cell viability assays (alamarBlue^®^, CellTiter-Glo^®^ and LDH) we have shown that all of our cell spheroid lines undergo a high amount of cell remodeling during the initial spheroid formation process within the first 72 h after seeding. We have identified three distinct phases of growth during the spheroid formation in all the cell lines in this study. Based on these results, we feel days 4–5 after seeding of cells is ideal for functional spheroid analysis.

The phenomenon of EMT involves features such as the gradual dissolution of cell–cell junctional connections to the adjacent cells, remodeling of the actin cytoskeleton and production of microenvironment matrix remodeling factors. The “stemness” of the cancer cells has been observed to be an important factor in determining the metastatic potential in liver cancer [15, 21]. While there are a multitude of molecular markers to determine the “stemness” or epithelial/mesenchymal nature of the cells, there is a relative paucity of observable physical or morphological features of the EMT phenomenon [16]. In the current study we have observed a distinct difference in the morphological features of the final spheroid configuration formed depending on the TP53 mutational status of the cell line. To our knowledge, this is the first instance of such a morphological descriptive difference observed in a 3-D tumor spheroid. The morphology in each cell line is consistent for the duration of the growth of the cell line over 10 days in multiple replicates of spheroid growth. These morphological differences are not immediately apparent in cell-culture flasks in two dimensions. It is interesting to speculate if these changes in the morphology may serve as a physical and mechanistic marker of the phenomenon of EMT. The variety of sizes and shapes observed in different cell lines indicates the sensitivity of cells to their microenvironment and illuminates the importance of specifically designed conditions in order to more accurately represent the in vivo model.

A number of molecular markers have been associated with the phenomenon of EMT [15]. A limited list of these markers involved in HCC are SNAIL1, SNAIL2, ZEB1, ZEB2, TWIST1, FOXF1, GATA4, GATA6, CDH1 (E-cadherin), β-catenin, Wnt signaling and CDH2 (n-cadherin) [15]. In the current paper, we chose CDH1 (E-cadherin) as a marker to explain the differing morphologies due to its central role in the formation of the adherens junctions. Indeed, we observed a significant difference in the expression patterns of the five cell lines depending on the TP53 mutational status. The TP53 wild type cell lines of HepG2 and C3A show a well-rounded spheroid morphology while the TP53 mutant (SNU-387 and SNU-475) show a smaller size with a rough boundary of edge growth. These morphologies were consistent with the relative lack of CDH1 expression levels which we demonstrated using immunofluorescence, real-time PCR and western blotting methods in the current study. However, the most intriguing observation was the overexpression of CDH1 protein in the TP53-null cell line, Hep3B. The overexpression of CDH1 in Hep3B leads to formation of relatively compact spheroids. An important note in this context of CDH1 gene expression in our chosen five cell lines is the presence of wild-type (no mutations) CDH1 gene sequences as determined from the publicly available database at the cancer cell lines encyclopedia (CCLE) of the Broad institute [17]. The lack of CDH1 mutations indicates that the expression and functionality of CDH1 in our five cell lines of the study is probably determined by other non-gene sequence level regulatory factors such epigenetic mechanisms or other signaling molecules. The regulation of CDH1 gene expression is complex and is driven by either promoter hypermethylation modulated by levels of enzymes such as DNMT1 and DNMT 3A/B or protein transcription factors such as ZEB1, ZEB2, SNAIL1, SNAIL2 and Twist [13]. Future studies in our lab aim to understand the precise mechanisms of CDH1 regulation driven by TP53 gene expression in these five cell lines in the 2-D and 3-D models. Evidence does exist for the regulation of CDH1 gene expression by TP53 protein levels in the context of ovarian cancer from a previous study [22].

In conclusion, we have established and characterized a method for 3-D spheroidal growth of liver cancer cell lines. We observe distinct morphological characteristics in the spheroid related to the TP53 mutational status of the cell line. CDH1 expression among our cell line panel is observed to be variable and distinct in the five cell lines of this study with maximal expression the TP53-null cell line, Hep3B. We propose that the morphological characteristics of the 3-D spheroids may serve as a physical proxy for the overall EMT status of a cell line.

## Methods

### Cell culture

C3A, HepG2, Hep3B, SNU-387 and SNU-475 liver cancer cell lines were obtained from American Type Culture Collection (ATCC, Manassas, VA). Individual HCC cell lines were chosen based on TP53 mutation status: HepG2 and its clonal derivative, C3A, express wild type TP53, Hep3B is TP53-null and the SNU-387 and SNU-475 cell lines express mutant TP53 protein. Cell stocks were maintained in T-75 flasks under standard culture conditions (5 % CO2, 37 °C), in either EMEM (wt/null cell lines; ATCC) or RPMI-1640 (Mutant cell lines; Thermo Scientific, Waltham, MA; 11875-101), supplemented with 10 % fetal bovine serum (FBS, Thermo Scientific, 16000077).

### Spheroid culture and imaging

HCC spheroids were formed using the hanging drop method with the aid of Perfecta3D^®^ Hanging drop plates (HDPs, 3DBiomatrix^®^, Ann Arbor, MI; HDP1096-8). After trypsinization, cells from culture flasks were centrifuged, resuspended and counted using a Countess Cell Counter (Life Technologies, Carlsbad, CA.). Cells were then seeded into 96-Well Perfecta3D^®^ HDPs (3D Biomatrix^®^; HDP1096-8) at a concentration of 6000 cells/well. Cells were supplemented with 1.5 % methylcellulose (MC) stock solution to aid in spheroid formation. 0.75 g of methylcellulose powder (Sigma-Aldrich, St. Louis, MO; M0512) were added to 50 mL sterile Phosphate Buffered Saline (PBS, GIBCO; 15517-022) and autoclaved. The resulting solution was centrifuged (5000*g*, 30 min, room temperature) and the supernatant was saved and aliquoted. 0.30 % Cultrex^®^ Basement Membrane Extract (BME, Trevgien, Waltham, MA; 3632-005-02) was also tested in the initial trials but discontinued in favor of MC. Suspended hanging drops contained a total volume of 40 µL according to company recommendations; 32 µL of the cell suspension contained the appropriate media (EMEM or RPMI). The remaining 8 µL was comprised of 1.5 % MC solution, resulting in a final concentration of 0.30 % MC per well. In later trials, the final concentration of MC was increased to 1 % for SNU-387 and SNU-475 with the expectation that it would improve spheroid compactness due to the increased viscosity of the medium. Small volume media droplets are prone to evaporation, so HDP humidity control was achieved by filling the plate reservoirs with a 0.5 % solution of LMP Agarose in PBS (autoclaved). This mixture created a resilient, gelatinous wall, preventing excessive evaporation. Media was changed by aspirating a small volume of the droplet (~ 5–10 µL) and adding between 10–20 µL of fresh media, resulting in a final volume of between 40–50 µL on alternate days. The spheroids were imaged using a Celestron Digital Microscope Imager (Celestron, Torrance, CA; 44421) at consecutive 24 h intervals for a duration of 10 days. To preserve the integrity of the sterile-HDP environment, spheroids were imaged from above with a 2.5×, wide-angle objective without removing the plate cover. Differences in spheroid diameter and shape were quantified using the open source software, ImageJ (NIH).

### Spheroid characterization assays

#### AlamarBlue^®^ Cell Proliferation Assay

To measure cell proliferation rates, we used a simple, one-step alamarBlue^®^ kit from AbDSerotec (AbD Serotec, Raleigh, NC; BUF012A). alamarBlue^®^ reagent was added to appropriate wells within the HDP at 10 % of the total volume of the droplet (4 µL in most cases). Reagent was also added to control wells containing media only (no spheroid) as a negative control. Cells were then incubated under standard conditions for 4 h. Absorbance values were obtained at 535 and 595 nm using a Perkin Elmer 1420-040 Victor 3 V plate reader. Reduction related to growth causes the redox indicator to change from oxidized (non-fluorescent, blue) form to reduced (fluorescent, red) form detected by the plate reader.

#### CellTiter-Glo^®^ luminescent viability assay

We used an alternative method, CellTiter-Glo^®^ (Promega, Madison, WI; G7570) which uses ATP and a luciferase-based substrate to indicate cell viability. Spheroids from each line were collected from the HDP into small volume eppendorf tubes at 1, 3, 5, 7 and 10 days incubation. CellTiter-Glo^®^ substrate was added by volume to each tube according to the manufacturer’s recommendations. The resulting solution was vortexed thoroughly to aid in cell lysis. After 15 min incubation at room temperature, 120 µL of solution was transferred from each tube into a LUMNITRAC 200 white-walled 96-well plate (USA Scientific, Ocala, FL; 5665-5075). Luminescence was measured using a Perkin Elmer Victor 3 V plate reader.

#### LDH cytotoxicity assay

To assess the cell death in the spheroid cultures, media samples of 15 µL were extracted in triplicate from each cell line (along with negative controls) at 1, 3, 5, 7 and 10 day time-points of spheroid growth. Each sample was transferred from the HDP into a LUMNITRAC 200 white-walled 96-well plate (USA Scientific; 5665-5075). LDH reagent was added according to manufacturer instructions. All samples were allowed to incubate for 30 min at room temperature before adding the reaction stop-solution. Sample absorbance was then measured at 490 and 575 nm using a Perkin Elmer Victor 3 V plate reader.

### 2-D immunofluorescence staining

Liver cancer cells were seeded onto circular, poly-d-lysine coated coverslips (Neuvitro, Vancouver, WA; GG-18-1.5-pdl) and incubated overnight in 6-well culture plates. Cells were washed with PBS and then fixed for 30 min in 4 % formalin (Sigma-Aldrich, St. Louis, MO; HT501128-4L). Cells were later incubated with D0-1 TP53 antibody (Santa Cruz, Dallas, TX; sc-126) or anti E-Cadherin antibody (Abcam, Cambridge, UK; ab76055), washed and then followed immediately by Alexa Fluor^**®**^ 488 secondary antibody (LifeTechnologies; A31620). Negative controls included both the addition of primary or secondary antibody alone. Coverslips were then washed thoroughly, coated with FluoroGel (Electron Microscopy Science, Hatfield PA; 17985-01) and fixed onto standard microscope slides. Slides were imaged at 63× using a Zeiss Axioskop.

### Quantitative real-time PCR (qRT-PCR)

RNA was isolated from both 2-D cell culture and 3-D spheroids using the PureLink^®^ RNA Mini Kit (Life Technologies; 12183018A). To maximize the amount of RNA from 3-D spheroids, each HCC cell line was seeded into 16 wells within the HDP. All spheroids were processed with the PureLink^®^ kit on day 4 after seeding. After the initial elution, a clean-up step using the RNeasy Mini Kit (Qiagen, Venlo, NL; N74104) was performed to improve RNA quality. cDNA was synthesized using SuperScript^®^ II Reverse Transcriptase (Life Technologies, 18064-022) according to manufacturer instructions. We elected to use custom oligo-sequences purchased from Integrated DNA Technologies (IDT, Coralville, IA) for TP53, CDH1 gene and the TATA Binding Protein (TBP) housekeeping control. qRT-PCR was performed in a Roche LightCycler^®^ 96 (Hold at 95 °C for 10 min, then 40 cycles of: 95 °C for 15 s and 60 °C for 1 min) and analyzed using the comparative cycle threshold method (2^−∆∆CT^) to quantify gene expression. The following RT-PCR probes were used for gene expression analysis in all of the samples examined in this study. TP53-alpha, Exon 9/10, Forward:5′-aaccactggatggagaatatttcac-3′, Reverse:5′-cagctctcggaacatctcgaa-3′, Probe:FAM-tcagatccgtgggcgtgagcg-VIC, CDH1, Exon6/7, Forward: 5′-ctgaggatggtgtaagcgatg-3′, Reverse:5′-gtctgtcatggaaggtgctc-3′, Probe: FAM-agacgcggacgatgatgtgaacac-VIC, Housekeeping gene, TBP (TATA Binding Protein), Forward:5′-cacgaaccacggcactgatt-3′, Reverse:5′-ttttcttgctgccagtctggac-3′, Probe: FAM-tgtgcacaggagccaagagtgaaga-VIC.

### Western blotting

Whole protein lysates from both 2-D and 3-D cultures were extracted using RIPA buffer under standard conditions. Protein concentrations were determined using the detergent-compatible, BCA Protein Assay Kit (Pierce, Grand Island, NY; 23227). Protein lysates were separated using SDS-PAGE electrophoresis and transferred to nitrocellulose membranes for probing. The monoclonal mouse antibody, DO-1 (SantaCruz; sc-126, dilution 1:200) was used to probe full-length p53 protein, while M168 antibody (Abcam, Cambridge, ab76055, dilution 1:200) was used to probe E-cadherin. The blots were then washed and incubated with a goat anti-mouse-HRP conjugate (SantaCruz; sc-2005, dilution 1:1000). After probing, blots were treated with either Amersham ECL Detection Reagent (GE Healthcare, RPN2209) or SuperSignal™ West Femto Maximum Sensitivity Substrate (ThermoFisher; 34095) and imaged using a Bio-Rad ChemiDoc™. Blots were then stripped and re-probed with GAPDH primary antibody (SantaCruz; sc-32233) as a positive loading control.

## Abbreviations

ECM: extra-cellular matrix
HCC: hepatocellular carcinoma
HDP: hanging drop plates
PBS: phosphate buffered saline
MC: methylcellulose
BME: basement membrane extract
LDH: lactate-dehydrogenase
IF: immunofluorescence
